# Editorial: Neuroscience in Africa

**DOI:** 10.3389/fnana.2019.00012

**Published:** 2019-02-14

**Authors:** Nilesh B. Patel, Nouria Lakhdar-Ghazal, Vivienne A. Russell

**Affiliations:** ^1^University of Nairobi, Nairobi, Kenya; ^2^Mohammed V University, Rabat, Morocco; ^3^University of Cape Town, University of KwaZulu-Natal, Cape Town, South Africa

**Keywords:** Africa, biodiveristy, HIV/AIDS, trypanosomiasis, fetal alcohol effects, emerging viral diseases, environmental pollutants

Reference to the brain and nervous system is found in 5,000-year-old Ancient Egyptian writings on medical procedures (Russell). In the twentieth century, modern neuroscience research began in Africa on potential treatments for epilepsy, infectious disease, nutritional neuropathies, stroke, and tumors. By the twenty-first century, African neuroscience expanded to other areas of basic and clinical neuroscience. This research topic on “Neuroscience in Africa” is a collection of reviews and original articles on the diversity of research being done in Africa. It represents studies on unique African fauna and flora, environmental pollution, neglected infectious diseases, and other neurological disorders—some of which are of global concern.

The diverse African fauna can potentially contribute to a better understanding of brain structure/function relationships. The black (*Diceros bicornis*) and the white (*Ceratotherium simum*) rhinoceros, amongst the largest terrestrial species on the African continent, have different behaviors: the black rhinoceros is solitary while the white rhinoceros is social. Both the black and white rhinoceros brains showed a typically mammalian organization and similar volumes of brain regions (Bhagwandin et al.). Congo African gray parrot (*Psittacus erithacus*) and Timneh gray parrot (*Psittacus timneh*) brains had widespread distribution of markers for adult neurogenesis, perhaps a reflection of their learning capacity and adaptation to environmental changes (Mazengenya et al.). In the Rock hyrax (*Procavia capensis*) REM sleep duration increased after being returned to a social environment following a period of isolation (Gravett et al.). The Dromerdary camel (*Camelus dromedaries*), changed cytoarchitecture and increased expression of oxytocin and tyrosine hydroxylase in its suprachiasmatic nucleus as it adapted to photic and non-photic cues under desert conditions (El Allali et al.).

Behavior in mice changed with exposure to glyphosate-based insecticide (Bali et al.), and exposure to paint thinner impaired neurodevelopmental processes (Malloul et al.). Regional accumulation and morphological abnormalities were seen after chronic vanadium administration and withdrawal in mice (Folarin et al.). Behavioral, electrophysiological, and neurochemical studies showed a role for noradrenaline depletion in lead-induced atypical parkinsonism in rats (Sabbar et al.). Chronic exposure to lead altered circadian clock proteins and the daily locomotor activity in rats (Sabbar et al.).

Sleeping sickness (human African trypanosomiasis), a neglected tropical disease, occurs in many sub-Saharan African countries. In the Natal multimammate mouse, *Mastomys natalensis*, infected with trypanosoma brucei gambiense, neural damage was seen in the suprachiasmatic nucleus and hypothalamic peptidergic sleep and wake-regulatory neurons (Laperchia et al.; Tesoriero et al.).

Cerebrospinal fluid amino acid profiling identified five elevated biomarkers which could be used to identify pediatric patients at an early stage of tuberculosis meningitis so facilitating early treatment (Mason et al.).

Cultural practices in some African countries result in high consanguinity. A combination of microarray with next-generation sequencing was used to identify genes involved in autosomal recessive Parkinson's disease among Moroccans (Bouhouche et al.). Moroccan patients with non-motor symptoms of Parkinson's disease had decreased quality of life (Tibar et al.), and Deep Brain Stimulation in treatment of Moroccan patients with Parkinson's disease showed a reduction in the most disabling motor symptoms and improved quality of life (Rahmani et al.).

Africa has unique ecosystems with high biodiversity and knowledge of its use in traditional medicine has a potential for discovery of novel therapeutic agents. Extracts of *Anacyclus pyrethrum* roots showed anti-inflammatory, antinociceptive, and antioxidant properties (Manouze et al.). *Pergularia daemia* had antiepileptogenic and neuroprotective effects in the pilocarpine model of epilepsy (Kandeda et al.). *Dichrocephala integrifolia* provided neuroprotection in the scopolamine mouse model of Alzheimer's Disease (Kouémou et al.). *Gladiolus dalenii* reduced stress-induced behavioral, neurochemical, and reproductive changes in rats (Fotsing et al.). Endocannabinoids produced analgesic effects in a mouse model of antiretroviral-induced neuropathic pain (Munawar et al.).

Emerging viral infections in Sub-Saharan Africa such as Ebola, West Nile, Zika, and Chikungunya cause brain malformations in prenatal infections and cognitive and psychiatric disturbances in perinatal or later infections (Kakooza-Mwesige et al.).

Africa has over 60% of the global HIV/AIDS infection. Despite early antiretroviral treatment, working memory and functional connectivity between networks was altered in 7-year-old HIV infected children (Milligan and Cockcroft; Toich et al.) and white matter abnormalities were seen (Jankiewicz et al.; Randall et al.). HIV-infected children had difficulty processing tasks with verbal or visuospatial modalities (Milligan and Cockcroft). Perinatal HIV infection or exposure was also associated with low N-acetylaspartate and glutamate in basal ganglia at age 9 but not at 7 (Robertson et al.).

Fetal alcohol syndrome rates in certain communities in South Africa such as the Western Cape Province are quite high. In children with prenatal alcohol exposure, decreased corpus callosum volume was associated with lower IQ (Biffen et al.), and impaired activation of parietal areas during non-symbolic number comparison (Woods et al.). In an animal study, off-spring of pregnant mice given alcohol, orexin-A neurons in the hypothalamus were larger and the density of orexinergic boutons in the anterior cingulate cortex was lower than controls (Olateju et al.).

Both reaction time and accuracy measures of intraindividual variability were found to predict cognitive performance in patients with Alzheimer's disease (Christ et al.). A mechanism of age-related synaptic dysfunction was proposed, based on the impact of IGF-1 receptor signaling on synaptic CaMKIIα phosphorylation (Ogundele et al.). Hippocampal neurodegenerative pathology did not differ between demented and non-demented post-stroke patients, however, tau immunoreactivity correlated negatively with memory scores (Akinyemi et al.). Children with severe traumatic brain injury showed poorer academic outcomes if accompanied by increased externalizing behavioral problems and executive dysfunction, and they were more likely to require special educational services (Dollman et al.). In a study of stress, no correlation was found between elevated cortisol levels and working memory in male and female volunteers (Human et al.).

The research presented in this research topic on “Neuroscience in Africa” gives a snapshot of the neuroscience research in Africa. It will be useful to those looking for research collaborations and information on African neuroscience relevant to their research areas. The support of the International Brain Research Organization (IBRO) and Society of Neuroscientists of Africa (SONA) is gratefully acknowledged.

## Author Contributions

VR prepared the initial draft of the editorial and NP, VR, and N-LG edited, reviewed and prepared the final submission.

### Conflict of Interest Statement

The authors declare that the research was conducted in the absence of any commercial or financial relationships that could be construed as a potential conflict of interest.

